# Metacognition and metacognitive monitoring in aging

**DOI:** 10.3758/s13423-026-02968-7

**Published:** 2026-08-04

**Authors:** Ali Pournaghdali, Gloria L. Cheng, Stephanie Cosentino, Teal S. Eich

**Affiliations:** 1 Miami Neuroscience Institute, Baptist Health South Florida, Miami, FL USA; 2 Leonard Davis School of Gerontology, University of Southern California, 3715 McClintock Avenue, Los Angeles, CA 90089 USA; 3 Taub Institute for Research on Alzheimer’s Disease and the Aging Brain, Columbia University Medical Center, New York, NY USA; 4 Department of Neurology, Columbia University Medical Center, New York, NY USA; 5 Gertrude H. Sergievsky Center, Columbia University Medical Center, New York, NY USA

**Keywords:** Aging, Metacognition, Metamemory

## Abstract

Metacognitive monitoring, or the capacity to assess and regulate one’s cognitive activities, is essential for maintaining optimal mental functioning. Metacognitive monitoring may be particularly important for individuals as they age, fostering self-awareness of cognitive strengths and limitations, guiding strategy selection to enhance performance, compensating for age-related changes to cognitive abilities, and identifying the earliest cognitive symptoms of disease. By evaluating one’s own understanding, individuals can align their confidence with actual ability, promoting better decision making and behavioral control. This review synthesizes the current literature on age-related differences in the accuracy of metacognitive monitoring across different types of metacognitive judgments, including judgments of learning, feelings of knowing, and post-decision confidence. We begin by discussing two key components of metacognitive monitoring: metacognitive sensitivity and metacognitive calibration. Then, we examine existing studies to address two main questions: whether age-related differences in one component (e.g., metacognitive calibration) can be explained by variations in the other (e.g., metacognitive sensitivity), and whether these differences can be attributed to age-related changes in underlying cognitive or perceptual processes. We find that the current literature does not provide definitive evidence for or against the decline of metacognitive monitoring in older adults. Rather, findings suggest that aging may differentially affect metacognitive accuracy depending on the type of judgment and task demands, with some domains showing preserved or even enhanced performance. These mixed findings underscore the conceptual and methodological complexity of studying metacognition in aging. We conclude by outlining key theoretical and empirical directions to clarify mechanisms and advance the field.

**Supplementary Information:**

The online version contains supplementary material available at https://doi.org/10.3758/s13423-026-02968-7.

## Introduction

Metacognition, defined as one’s knowledge concerning one’s own cognitive processes and products or anything related to them (Gurat & Medula, 2016, p. 171; also see Koriat, 2007, 2015; Metcalfe & Schwartz, 2016), is an important cognitive ability across the lifespan, but may be particularly consequential in later life. As individuals grow older, many cognitive abilities begin to decline (Balota et al., 2000; Cabeza et al., 1997; Craik, 1994; Habeck et al., 2018), making metacognitive monitoring, or the process of judging one’s own cognition, crucial for adapting to these shifts and maintaining optimal mental functioning. For example, if an older adult recognizes their inability to remember daily medicine dosages, they may adopt compensatory strategies, such as using a pill organizer (Baltes & Baltes, 1990) or an external memory aid to offload important information (Burnett & Richmond, 2023; Murphy & Castel, 2023). In this way, metacognition facilitates self-awareness of cognitive strengths and weaknesses, aiding in the selection of appropriate strategies to enhance cognitive performance and compensate for age-related declines (Hertzog, 2016). Beyond everyday functioning, it is also thought to play a central role in early subjective detection of neurodegenerative diseases such as Alzheimer’s disease (Chapman et al., 2020; Cosentino, 2014; Cosentino et al., 2007; Sunderaraman & Cosentino, 2017), before such symptoms are observable through objective cognitive testing (i.e., Subjective Cognitive Decline (SCD); Cosentino et al., 2011).

Metacognitive processes further contribute to effective decision making, problem solving, and learning, crucial components for maintaining independence and quality of life in later adulthood (Dunlosky & Metcalfe, 2009). Moreover, metacognition plays a crucial role in maintaining mental health and emotional well-being by facilitating recognition and regulation of cognitive distortions and negative thought patterns (Dixon & Hultsch, 1983). By fostering a proactive approach to cognitive health, metacognition may support adaptive responses to cognitive and physiological challenges in later life and encourage continued engagement in learning and cognitively stimulating activities.

The present review focuses on age-related differences in metacognitive accuracy, defined as the degree to which an individual’s subjective judgments about their performance corresponds to their actual objective performance (Fleming & Lau, 2014; Nelson, 1984). Although metacognitive accuracy is highly relevant to pathological aging and neurodegenerative conditions, our emphasis here is on “normal,” non-pathological aging (for reviews of both metacognitive monitoring and accuracy in clinical populations, see Bertrand et al., 2016; Chapman et al., 2020; Latgé-Tovar et al., 2022; Rosen et al., 2014; Souchay & Moulin, 2009; Starkstein, 2014; Sun et al., 2022; Wilson et al., 2016).

We begin by examining how metacognitive accuracy can be assessed in general. We then review studies that have evaluated different types of metacognitive judgments that index metacognitive monitoring, the process of generating metacognitive judgments about one’s own performance, with a focus on studies that have explored these processes in both younger and older adults. Finally, we identify key gaps in current understanding of how aging affects metacognitive accuracy and propose several directions for future research to address these gaps.

### How do we assess metacognitive accuracy?

Metacognitive accuracy refers to the extent to which an individual’s subjective judgments about their cognitive performance corresponds to their actual performance. In laboratory studies, metacognitive accuracy is often assessed by asking participants to make explicit metacognitive judgments about their decisions, memories, or perceptions. For example, imagine participants study face-name pairs for a subsequent memory test. Objective accuracy is assessed by presenting each face with either its originally studied name (intact) or a novel name (rearranged) and asking participants to decide whether the pair is intact or rearranged. Metacognitive monitoring is assessed by asking participants to rate their confidence in the accuracy of each decision. For this task, we can calculate two distinct levels of performance: objective memory performance (e.g., discrimination between intact and altered pairs) and metacognitive accuracy, reflecting how well confidence ratings differentiate correct from incorrect responses. Within this metacognitive level, performance can further be distinguished into metacognitive sensitivity (also referred to as metacognitive resolution or relative accuracy; Fleming & Lau, 2014; Nelson, 1984) and metacognitive calibration (also referred to as absolute accuracy; Pieschl, 2009; Stone, 2000).

In this example, the first component of metacognitive accuracy is metacognitive sensitivity, which is traditionally defined as the extent to which metacognitive judgments can accurately predict and discriminate between correct and incorrect object-level responses (Fleming & Lau, 2014; Nelson, 1984). High metacognitive sensitivity would mean that confidence ratings reliably discriminate correct from incorrect responses, whereas low metacognitive sensitivity would mean that confidence fails to make this distinction.

In the aging literature, metacognitive sensitivity is most commonly measured using within-subjects rank-order gamma (γ) correlations (Goodman & Kruskal, 1954; Nelson, 1984), which quantifies the ordinal association between metacognitive judgments and object-level task performance accuracy. A positive γ indicates that a metacognitive judgment reliably predicts object-level accuracy, whereas a chance-level γ (γ = close to 0) reflects the inability of a metacognitive judgment in predicting object-level accuracy. Although rare, a negative γ implies that a metacognitive judgment mis-predicts object-level accuracy (Benjamin et al., 1998).

In addition to γ, measures of metacognitive sensitivity based on an extension of signal detection theory (SDT; Green & Swets, 1966; Macmillan & Creelman, 2005) called type-2 signal detection theory (type-2 SDT; see Fleming & Lau, 2014) have been proposed: type-2 sensitivity (a’; Benjamin & Diaz, 2008; Kunimoto et al., 2001), type-2 receiver operating characteristic curve (type-2 ROC curve; Galvin et al., 2003; Masson & Rotello, 2009), and meta-d’ (Maniscalco & Lau, 2012, 2014). The last measure, meta-d′, is expressed on the same scale as object-level sensitivity (d’), which is an index for the ability of an individual to discriminate between different stimulus categories (e.g., intact vs. altered face-name pairs). Hence, we can normalize meta-d′ by dividing it by object-level sensitivity. This normalized index, M-ratio, represents the efficiency of a metacognitive judgment to utilize object-level evidence to evaluate object-level decisions.

Research indicates that type-2 sensitivity and type-2 ROC curve are influenced by both metacognitive calibration and object-level performance (e.g., Rahnev, 2023). As a result, these two SDT-based measures may not provide an exclusive estimate of metacognitive sensitivity. Although the M-ratio is not influenced by object-level effects, evidence suggests that metacognitive calibration may systematically influence the level of meta-d′ and, consequently, the level of M-ratio.

Other measures of metacognitive sensitivity include the V-ratio (Desender et al., 2022), log-normal meta-noise (Shekhar & Rahnev, 2021), meta-uncertainty (Boundy-Singer et al., 2023), sensitivity versus metacognition curve (Pournaghdali et al., 2024), and confidence noise (Mamassian & de Gardelle, 2022). However, these newer measures have not yet been used to examine age-related variation in metacognitive sensitivity, and thus we will not discuss them further.

Metacognitive sensitivity can be dissociated from metacognitive calibration, which refers to the extent to which overall confidence levels are appropriately matched to objective task performance (e.g., being over- or under-confident) (Glenberg & Epstein, 1987; Schraw & Roedel, 1994; Stone, 2000). Metacognitive calibration can be evaluated by asking participants to estimate the percentage of items they answered correctly across a series of items, or it can be computed at the item level by collecting confidence judgments for each individual response. In both cases, participants are considered well calibrated when their perceived accuracy corresponds closely to their observed accuracy. For example, if a participant believes they got 75% of the face-name pairs correct, and they got 75% correct, they are calibrated. In contrast, a lack of metacognitive calibration, referred to as metacognitive bias, arises when perceived performance deviates from actual performance. Under-confidence reflects a conservative metacognitive bias in which participants perform better than they thought they did, whereas over-confidence reflects a liberal metacognitive bias, in which participants believe they performed better than they actually did. Because metacognitive calibration and metacognitive bias represent complementary aspects of the same construct, metacognitive calibration can be evaluated by examining the degree of bias. High metacognitive calibration corresponds to low metacognitive bias, whereas low metacognitive calibration indicates greater metacognitive bias.

One straightforward approach to estimating metacognitive calibration is to calculate each participant’s average metacognitive judgment across trials. Under this approach, a participant with an average ranking close to the highest level of metacognitive judgment is interpreted as exhibiting a more liberal bias than a participant with an average ranking close to the middle level of that metacognitive judgment. This approach, however, has limitations, as the criterion used to define calibration or bias is often ambiguous. Moreover, metacognitive sensitivity can influence the average metacognitive ratings, thereby confounding estimates of metacognitive calibration (see Xue et al., 2021).

Another way of evaluating metacognitive calibration is to plot the proportion of correct object-level responses on the y-axis, against different ratings of a metacognitive judgment (e.g., 0, 10, 20, 30, …, 90, 100% confidence ratings) on the x-axis. Here, for each value of metacognitive judgment, the corresponding proportion of correct object-level decisions is estimated. This can be used to produce a confidence-accuracy calibration (CAC; see Weber & Brewer, 2004), where accuracy is plotted as a function of confidence, with a line fitted through the resulting points. A perfectly diagonal CAC curve represents optimal metacognitive calibration. Deviations from the diagonal line reflect metacognitive bias, with points or segments above the diagonal indicating under-confidence, and those below the diagonal indicating over-confidence.

Because the CAC curve does not provide a point-estimate of metacognitive calibration, the point-estimate can be determined by using either the calibration index or the over-/under-confidence index (e.g., Hertzog et al., 2002). Both indices quantify the difference between a participant’s average metacognitive ratings and their average object-level performance. The calibration index, however, provides an overall estimation of the deviation from the optimal metacognitive judgment without any information about the direction of bias (conservative or liberal), with 0 representing perfect calibration and 1 representing maximal bias. The over-/under-confidence index, on the other hand, estimates the magnitude and direction of deviation, with 0 representing perfect calibration, ˗1 representing complete under-confidence (conservative bias), and +1 representing complete over-confidence (liberal bias). Like the CAC curve, both indices are influenced by object-level task performance. Consequently, variations in task accuracy, as well as factors that affect object-level performance, can systematically affect estimates of metacognitive calibration derived from these measures.

Metacognitive calibration can also be evaluated within the framework of type-2 SDT (Charles et al., 2020). This approach involves first determining an optimal metacognitive criterion for each stimulus category, which maximizes the probability of making a type-2 hit (that is, a high metacognitive judgment to a correct object-level decision). Next, a subjective metacognitive criterion is estimated for each participant and for each category. Metacognitive calibration is then quantified by comparing a participant’s subjective criterion to the optimal criterion: the closer a participant’s actual criterion is to the optimal, the higher their calibration. Conversely, as a participant’s actual metacognitive criterion moves away from the optimal metacognitive criterion, the level of metacognitive bias increases (Charles et al., 2020). This formalization of metacognitive calibration and bias provides a more precise measure than the CAC curve or the calibration and over-/under-confidence indices. Nevertheless, object-level task performance can systematically influence the estimation of metacognitive sensitivity resulting from this approach (for a solution to these two problems, see Pournaghdali et al., 2024).

Metacognitive sensitivity and calibration are related yet dissociable components of metacognitive accuracy. For example, a participant might estimate that they answered 75% of face-name pairs correctly and actually achieve 75% correct. However, they might be incorrect about *which* items they got right and wrong. This person would be perfectly calibrated but have low sensitivity. On the other hand, a participant could assign higher confidence to correct responses than to incorrect responses on every trial, thereby achieving perfect sensitivity. However, if they systematically use extreme confidence values (e.g., reporting 100% confidence on correct trials and 60% confidence on incorrect trials), their confidence levels would not accurately reflect the true probability of being correct. In this case, sensitivity would be high, but calibration would be poor. Thus, differences in how participants use the rating scale can produce dissociations between metacognitive sensitivity and calibration.

Therefore, accurate evaluation of age-related differences in metacognitive processes depends on our ability to dissociate these two components of metacognitive accuracy and to provide an exhaustive estimation of each. Hence, we must ensure that the age-related differences in metacognitive sensitivity are not due to changes in metacognitive calibration, and, on the flip side, that age-related differences in metacognitive calibration are not due to changes in metacognitive sensitivity. This is particularly important given that different neurocognitive mechanisms may underlie the sensitivity and calibration of a metacognitive judgment (Shekhar & Rahnev, 2018; Xue et al., 2021). For example, metacognitive sensitivity of confidence judgments and metacognitive calibration of confidence may be supported by distinct regions in the frontal cortex (e.g., Behrendt et al., 2024; Fleming et al., 2010; Shekhar & Rahnev, 2018). Accordingly, both healthy and pathological aging may differentially influence these aspects of metacognitive accuracy (e.g., Dodson et al., 2007; Hertzog et al., 2002; Pliske & Mutter, 1996). To understand the specific impacts of healthy aging on metacognition, it is therefore critical to dissociate sensitivity from calibration (also see Evans & Azzopardi, 2007; Ko & Lau, 2012).

It is, of course, equally important to distinguish age-related differences in metacognitive sensitivity and calibration from age-related differences in overall task performance (McNicol, 2005). In our face-name learning task example, an older adult who can discriminate between intact pairs and rearranged pairs has high object-level sensitivity, whereas an older adult who cannot make this distinction has low object-level sensitivity.

Regardless of object-level sensitivity, participants may tend to choose one option more often than the other when asked to make a decision about two stimulus categories. For example, one older adult may tend to endorse most face-name pairs as “correct,” even when they are rearranged. Such biases, referred to as object-level bias, may arise from shifts in decision criterion driven by prior expectations, asymmetric perceived costs of errors, or strategic reliance on default responses under uncertainty – factors that may become especially pronounced in older adulthood even when underlying discriminability remains intact (McNicol, 2005; also see Azzopardi & Cowey, 1997; Heeks & Azzopardi, 2015; Pournaghdali et al., 2023).

Aging is associated with changes in basic task performance, including reduced perceptual sensitivity, changes in memory, and shifts in response bias (e.g., Devaluez et al., 2023). These differences can influence estimates of metacognitive sensitivity and calibration, even when metacognitive processes themselves are intact (e.g., Galvin et al., 2003; Rahnev, 2023). For example, if older adults perform near chance because they cannot reliably distinguish rearranged from intact pairs (Yassa & Stark, 2011), their confidence ratings cannot meaningfully track accuracy. In such cases, low metacognitive sensitivity may reflect degraded primary task performance rather than impaired metacognitive sensitivity (see also Maniscalco & Lau, 2012).

Therefore, metacognitive sensitivity in older adults may be systematically underestimated when age-related differences in task performance are not accounted for. This confounding makes it difficult to determine whether an observed age effect in metacognitive sensitivity reflects true differences in metacognitive processes or simply age-related cognitive changes. As a result, apparent age-related differences in metacognitive sensitivity may be exaggerated, attenuated, or even obscured, depending on how task performance and confidence judgments are related. A similar concern applies to metacognitive calibration. Therefore, when evaluating age-related differences in metacognitive sensitivity and calibration, it is essential to ensure that any observed results are not driven by age-related differences in task performance.

In summary, accurately evaluating age-related changes in metacognitive accuracy requires distinguishing between metacognitive sensitivity from metacognitive calibration, as well as separating age-related differences in these components from age-related differences in primary task performance. Addressing these distinctions will be a central focus of the remainder of this review.

### Different types of metacognitive judgments

The aim of this review is to evaluate the current literature on the impact of aging on metacognitive accuracy, considering both sensitivity and calibration, across different types of metacognitive judgments. We examine whether prior studies were able to distinguish age-related differences between metacognitive sensitivity and metacognitive calibration, and whether they adequately discriminate the age-related differences in these metacognitive effects from age-related differences in object-level sensitivity and object-level bias (for a general summary of the studies examining metacognitive sensitivity and calibration in older adults, see Table 1).

**Table 1:**
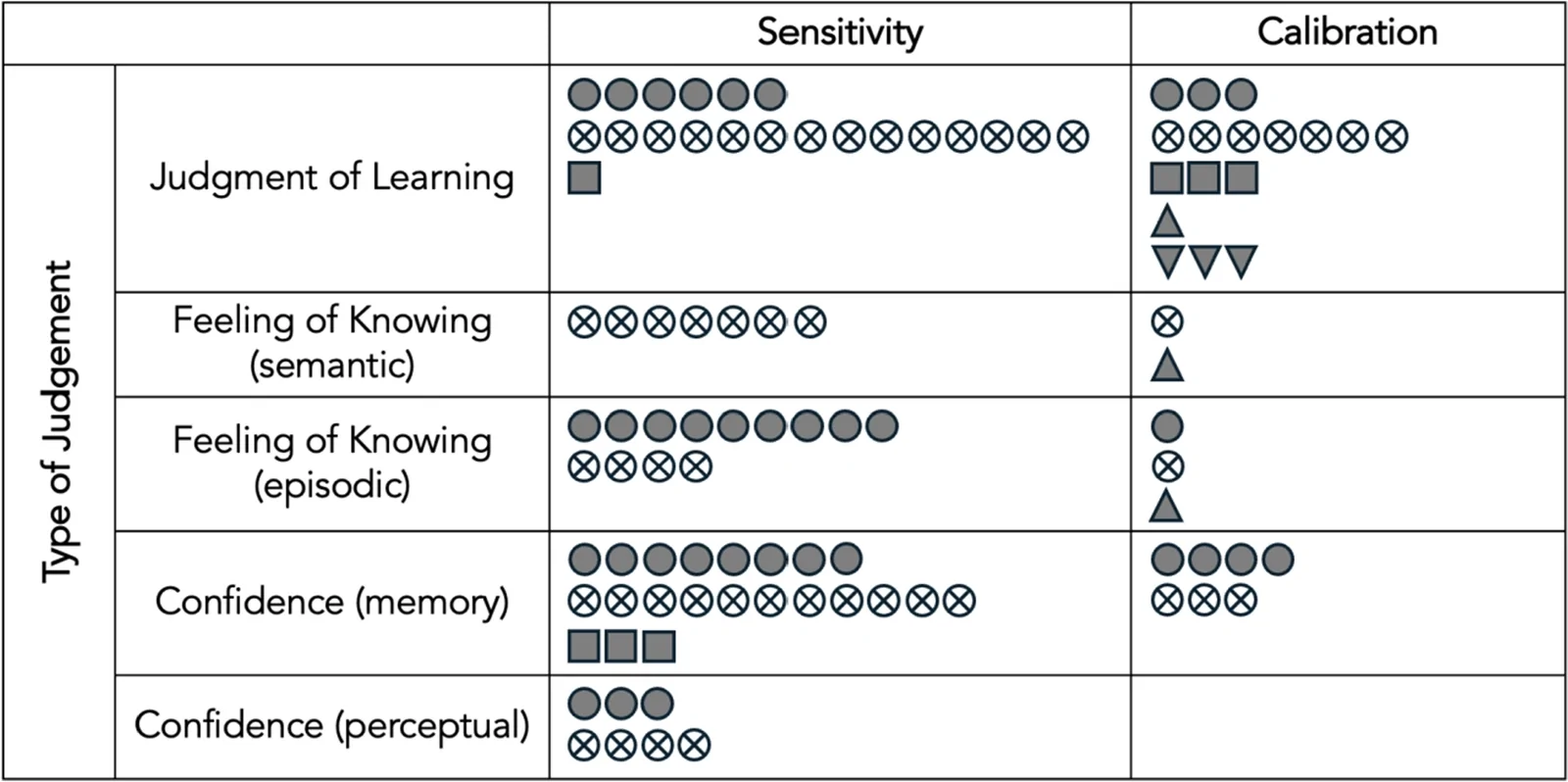
Summary of studies examining age-related differences in metacognitive sensitivity and calibration. Symbols represent study outcomes: solid circles indicate better performance in younger adults, solid squares indicate better performance in older adults, open circles with an “x” indicate no age-related difference, upward triangles indicate conservative bias in older adults, and downward triangles indicate liberal bias in older adults

### Judgments of learning (JOLs)

One of the most common types of metacognitive judgment is the Judgment of Learning (JOL), which assesses the metacognitive experience of learning by asking participants to rate the likelihood of being able to retrieve newly learned information (Bjork et al., 2013; Nelson & Dunlosky, 1992; Rhodes & Tauber, 2011). For example, after memorizing face-name pairs, participants rate the probability that they will be able to retrieve the correct name when presented with the face. A high JOL indicates that a participant believes that they are very likely to retrieve the associated name, while a low JOL indicates a lower perceived likelihood of retrieval. In general, JOLs are classified into two types: immediate-JOLs, which are reported immediately after participants study the to-be-remembered information, and delayed-JOLs, which are reported after a delay (Nelson & Dunlosky, 1992). Research indicates that delayed-JOLs, but not immediate-JOLs, more accurately predict subsequent memory performance (e.g., Nelson & Dunlosky, 1991, 1992).

### JOL sensitivity in older adults

Given there are age-related impairments in most cognitive processes, including episodic memory (Balota et al., 2000; Cabeza et al., 1997; Cargin et al., 2007; Corlier & Eich, 2023; Craik, 1994; Eich et al., 2017; Mitchell et al., 2000; Schacter, 1999), and binding features of episodic memories in particular (for a review, see Hertzog & Shing, 2011), evaluating metacognitive sensitivity of JOLs (henceforth, JOL sensitivity) is of critical importance. If older adults have a comparable or even superior JOL sensitivity relative to younger adults, they can use the results of their JOLs to compensate for possible impairment in episodic memory retrieval. For example, if an older adult predicts correctly that they are likely to forget their doctor’s recommendation about performing a specific type of physical activity, they can ask their doctor for written instructions or a catalog that explains that specific physical activity. Therefore, JOL sensitivity may play an important role in supporting older adults’ day-to-day cognitive functioning.

Table 2 shows a summary of studies that evaluated age-related differences in JOL sensitivity. Early research indicated that older adults have an intact level of immediate (Connor et al., 1997; Dunlosky & Hertzog, 2000; Hertzog et al., 2002, Experiment 2; Hines et al., 2009; Rabinowitz et al., 1982; Robinson et al., 2006; Shaw & Craik, 1989; but see Hertzog et al., 2002, Experiment 1), as well as delayed (Connor et al., 1997) JOL sensitivity. More specifically, age invariance in immediate-JOL sensitivity is due to older adults’ reliance on associate relatedness (Hertzog et al., 2002) and encoding fluency (Robinson et al., 2006), which are critical for the creation of JOL experiences (Hertzog et al., 2003; Koriat, 1997; also see Halamish & Undorf, 2023; Matvey et al., 2006; Schwartz et al., 2020).

**Table 2:** Summary of studies that evaluated age-related differences in judgment-of-learning (JOL) sensitivity

Study	Experiment	Task	Measure of sensitivity	Result	Young		Old	
					*N*	Age, y	*N*	Age, y
Connor et al. (1997)	1	Cued-recall	Gamma	Young = Old	60	20.2	34	71.1
	2				80	21.0	78	69.4
	3				30	20.1	30	65.5
Dunlosky and Hertzog (2000)	1	Cued- recall	Gamma	Young = Old	40	19.2	40	69.7
Hertzog et al. (2002)	1	Cued-recall	Gamma	Young > Old**	52	20.4	50	68.8
	2			Young = Old	37	19.8	31	69.5
Hines et al. (2009)	1	Cued-recall	Gamma	Young = Old	39	21.8	35	66.8
Robinson et al. (2006)	1	Cued-recall	Gamma	Young = Old	44	20.33	28	70.24
	2				47	19.38	29	70.81
Shaw and Craik (1989)	1	Cued-recall	Average JOL ratings for recalled vs. unrecalled pairs	Young = Old	18	19.4	18	68.7
Sanders and Berry (2021)	1	Cued-recognition of emotional words	Gamma	Young = Old	42	19.36	43	73.95
Sun and Jiang (2023)	1	Cued-recognition of emotional pictures	Gamma	Young = Old	29	21.55	32	64.66
Tauber and Dunlosky (2012)	1	Emotional words recollection	Gamma	Young > Old	31	20.4	30	69.2
	2			Young = Old	66	19.53	59	67.79
Tauber et al. (2017)	1	Emotional pictures recollection	Gamma	Young = Old	42	19.8	40	68.2
	2				38	20.2	42	71.0
Hertzog, Sinclair, et al. (2010b)	1	Cued-recall	Gamma	Young < Old***	100	29.62	92	68.57
Daniels et al. (2009)	1	Cued-recollection	Gamma	Young = Old Young>Old****	76	19.6	78	71.4
Thomas et al. (2012)	1a	Yes/no recognition	Gamma	Young = Old	24	21.3	24	74.0
	1b			Young > Old	24	19.3	24	74.3
	2			Young > Old	24	19.9	24	74.1
Toth et al. (2011)	1	Cued-recollection	Gamma Type-2 ROC Curve	Young = Old Young>Old*****	36	20.3	36	73.1
Rabinowitz et al. (1982)	1	Cued-recall	Average JOL ratings for recalled vs. unrecalled pairs	Young = Old	24	19	24	69

*This study evaluated age-related changes in delayed as well as immediate JOL sensitivity

** In this study, when considering related and unrelated pairs separately, age had a significant effect. However, when estimating JOL sensitivity using the aggregated Gamma, JOL sensitivity was the same between the two age-groups

***In this study, when considering related and unrelated pairs separately, age did not have a significant effect. However, when estimating JOL sensitivity using the aggregated Gamma, JOL sensitivity increased as a function of increase in age

**** In these studies, difference criteria used to estimate two different Gamma coefficients. The main text provides a more detailed description of the two criteria and the results

Further studies using stimuli with emotional valence similarly support the absence of age-related differences in JOL sensitivity (Sanders & Berry, 2021; Sun et al., 2022; Tauber et al., 2017; Tauber & Dunlosky, 2012, Experiment 2; but see Tauber & Dunlosky, 2012, Experiment 1). For example, Tauber and Dunlosky (2012, Experiment 2) asked younger and older participants to memorize a series of words that varied in emotional valence. Participants were assigned to one of three conditions: positive (e.g., bunny) and neutral (e.g., fabric) words, negative (e.g., cancer) and neutral words, or a mixed list of positive, negative, and neutral words. After studying each word, participants provided a JOL, followed by a free-recall test. Analysis of γ correlation revealed age-invariant JOL sensitivity. Across all conditions, both younger and older adults demonstrated above-chance JOL sensitivity, regardless of stimulus valence.

Other studies, however, have found age-related differences in JOL sensitivity. Hertzog, Dunlosky et al. (2010a) and Hertzog, Sinclair et al. (2010b)) asked participants aged 18 to 81 years to study related and unrelated cue-target pairs, provide JOL ratings after each pair, and recall the target word when presented with the corresponding cue. JOL sensitivity was evaluated with γ correlations, with function of age treated as a continuous variable. Although separate analysis of γ coefficients for related and unrelated pairs did not yield a significant main effect of age, the results of the aggregated γ coefficient indicated a significant enhancement of JOL sensitivity as a function of age. That is, as age increased, so too did JOL sensitivity.

Other studies show that older adults, under certain conditions, have lower JOL sensitivity compared to younger adults (Daniels et al., 2009; Tauber & Dunlosky, 2012, Experiment 1; Thomas et al., 2012, Experiments 1b & 2; Toth et al., 2011). For example, Daniels et al. (2009) asked older and younger adults to memorize a series of words and to provide a JOL rating immediately after each word. Next, they asked participants to retrieve the studied words using a memory recognition (categorize the words as “recollect,” “familiar,” or “no memory”) or a cued-recall task followed by a recognition test (first, complete the word stem, e.g., CLO--, even if the word could not be remembered, and then classify the completed word as “recollect,” “familiar,” or “no memory”). Two γ coefficients were estimated for each participant. The first considered “recollect” and “familiar” responses as recalled and “no memory” as unrecalled memory responses.^1^ The second used “recollect” responses as recalled and “familiar” and “no memory” responses as unrecalled memories.^2^ There were no age differences in the first γ coefficient across memory test types. However, the second γ coefficient, which was restricted to “recalled” memory responses, revealed lower JOL sensitivity in older adults than in younger adults. These findings suggest that age-related impairments in JOL sensitivity may emerge only when memory performance is defined in terms of recollection, which requires more evidence than familiarity judgments (see Hertzog & Dunlosky, 2011). Consequently, the observed age-related differences of JOL sensitivity in Daniels et al. (2009) may reflect differences in memory processes, rather than inefficiencies in JOL processes.

Support for this interpretation comes from Toth et al. (2011), who asked participants to study actor names from the 1950s and 1990s and provide immediate JOL ratings on the probability of remembering each name in a future test. At test, participants completed a memory recognition task, in which they categorized studied and novel actor names^3^ as “recollect,” “know,” and “no memory.” As in Daniels et al. (2009), two types of JOL sensitivities were estimated: one in which both “recollect” and “know” responses were considered as recalled memories, and another in which only “recollect” responses were considered as recalled memories. Importantly, Toth et al. (2011) estimated JOL sensitivity using the γ coefficient as well as the area under the type-2 ROC curve (${d}_{a}$; see Benjamin & Diaz, 2008).^4^ When using “recollect” and “know” as recalled memory responses, both γ coefficient and ${d}_{a}$ were comparable between younger and older adults. However, when using “recollect” as a recalled memory response, ${d}_{a}$ was lower in older adults than in younger adults, whereas the γ coefficient was lower in younger adults than in older adults when estimated for all the names, regardless of the decade.^5^ Together, the results of Daniels et al. (2009) and Toth et al. (2011) suggest that age-related differences in JOL sensitivity may depend on the criteria used to separate correct from incorrect memory decisions. Hence, such age effects may be due to age-related differences in object-level bias, and not age-related changes in JOL sensitivity.

In addition, Tauber and Dunlosky (2012, Experiment 1) found lower JOL sensitivity in older adults when monitoring memory for words with positive (e.g., fame), negative (e.g., cancer), or neutral (e.g., habit) valence. A follow-up experiment, however, failed to replicate this age-related deficit in sensitivity and reported that the observed effect may be due to reduced cognitive resources in older adults rather than deficits in JOL processes. Additionally, Thomas et al. (2012) showed age-related differences in JOL sensitivity in a visual working memory task. In their first experiment, younger and older adults studied 5 × 5 grids containing two to five objects presented in random locations. Younger adults were given 500 ms to study each grid, whereas older adults had 3,000 ms. Following each study phase, participants provided a JOL and completed a yes/no recognition task, in which they were asked about one of the following: object-identities (“Was this shape presented in the previous grid?”), object-location (“Was an object presented in this location in the previous grid?”), or object-location and identity (“Was this object presented in this location in the previous grid?”). The results showed age-invariance in JOL sensitivity. However, in the follow-up experiments, when encoding time was equated across age groups (1,500 ms; Experiments 1b and 2), younger adults demonstrated higher JOL sensitivity than older adults. The combination of results across this series of experiments highlights the role of age-related differences in object-level processing decisions, such as processing speed, and their downstream consequences for metacognitive accuracy (Hilton et al., 2020). Here, providing older adults with additional encoding time relative to younger adults rescued JOL sensitivity.

In summary, the majority of studies to date report age-invariant JOL sensitivity. However, under specific task conditions, older adults may have higher or lower JOL sensitivity than younger adults. Importantly, most studies of JOL sensitivity in older adults have used γ correlations as the measure of metacognitive sensitivity. Because γ is vulnerable to object-level differences and metacognitive bias, these factors may have contaminated and biased estimates of age-related differences in JOL sensitivity.

### JOL calibration in older adults

In addition to JOL sensitivity, a substantial body of work has evaluated JOL calibration in older adults (see Table 3 for a summary). The results of these studies paint a mixed picture of age-related differences. Early studies indicated age-invariance in JOL calibration (Dunlosky & Hertzog, 2000; Hertzog et al., 2002, Experiment 2; Rabinowitz et al., 1982; but see Shaw & Craik, 1989). In contrast, Connor et al. (1997) found a different age-related pattern of results across a series of experiments. In their first experiment, participants provided immediate JOLs for some word pairs and delayed JOLs for other pairs. Older adults were less calibrated and more liberally biased, characterized by overconfidence, for both immediate and delayed JOLs. In their second experiment, participants studied each word pair twice to enhance recollection. This time, older adults demonstrated better calibration for both immediate and delayed JOLs. Finally, in their third experiment, semantic association between word pairs was manipulated to ensure participants had at least intermediate recall performance. With this manipulation, older and younger adults showed similar calibration for immediate JOLs. In the delayed-JOL condition, older adults showed signs of calibration, though they were less calibrated than younger adults. Taken together, these studies suggest that older adults’ JOL calibration may depend on memory strength. By intervening in the strength of memory processes, either through repeated study or semantic support, older adults may adopt more optimal JOLs and enhance their metacognitive calibration. However, visual inspection of the confidence-accuracy calibration (CAC) curve^6^ across all three experiments reveals an interesting age-related pattern: regardless of the overall level of calibration, older adults deviate from optimal calibration at higher levels of JOL judgment. This observation warrants further empirical consideration.

**Table 3:** Summary of studies that evaluated age-related differences in judgment-of-learning (JOL) calibration

Study	Experiment	Object-level task	Measure of iInterest	Result	Young		Old	
					*N*	Age, y	*N*	Age, y
Connor et al. (1997)*	1	Cued-recall	CAC Curve	Older adults Lower calibration and liberal bias	60	20.2	34	71.1
	2			Older adults Better calibration	80	21.0	78	69.4
	3			Immediate-JOL Young = Old Delayed-JOL Older adults Lower calibration	30	20.1	30	65.5
Dunlosky and Hertzog (2000)	1	Cued-recall	Over-/under-confidence index	Young = Old	40	19.2	40	69.7
Hertzog et al. (2002)	1	Cued-recall	Calibration index	Older adults Better calibration	52	20.4	50	68.8
			Over-/under-confidence index	Younger adults Conservative bias				
	2	Cued-recall	Calibration index	Young = Old	37	19.8	31	69.5
			Over-/under-confidence index	Older adults Liberal bias Younger adults Conservative bias				
	3	Cued-recall	Calibration index	Older adults Lower calibration	119	19.2	49	70.2
			Over-/under-confidence index	Younger adults Conservative bias				
Robinson et al. (2006)	1	Cued-recall	Average JOL Ratings	Younger adults Conservative bias Older adults Liberal bias	44	20.33	28	70.24
	2				47	19.38	29	70.81
Shaw and Craik (1989)	1	Cued-recall	Average JOL Ratings	Older adults Liberal bias	18	19.4	18	68.7
Hertzog, Sinclair et al. (2010b)	1	Cued-recall	Calibration index	Older adults Better calibration**	100	29.62	92	68.57†
Thomas et al. (2012)	1a	Identity recognition	Over-/under-confidence index	Older adults Conservative bias	24	21.3	24	74.0
		Location recognition		Young = Old				
		Identity/location recognition		Young = Old				
	1b			Young = Old	24	19.3	24	74.3
	2			Young = Old	24	19.9	24	74.1
Rabinowitz et al. (1982)	1	Cued-recall	Average JOL Ratings	Young = Old	24	19	24	69

*This study evaluated age-related changes in delayed as well as immediate JOL sensitivity

**In this study, older adults were more calibrated in all conditions except when they reported using encoding strategies for related word-pairs, which resulted in a lower calibration in older adults

† This study also included mid-life adults (*N *= 92, mean age = 49.6 years)

In contrast to Connor et al. (1997), Hertzog et al. (2002, Experiment 1) showed that older adults are more calibrated than are younger adults when participants studied related and unrelated word pairs and provided immediate JOLs. Comparing calibration scores of older and younger adults in follow-up experiments, however, revealed that either the age groups had similar levels of metacognitive calibration (Experiment 2) or that younger adults were more calibrated than older adults (Experiment 3). Across all three experiments, metacognitive calibration was also calculated using the over-/under-confidence index, which represents the degree and direction of deviation from optimal calibration. Younger adults consistently showed conservative bias, underestimating their actual recall performance (for a similar result, see Robinson et al., 2006,7). The results align with other cross-sectional studies by Hertzog, Dunlosky et al. (2010a) and Hertzog, Sinclair et al. (2010b), which showed that older adults had better JOL calibration for unrelated word pairs. However, for related word pairs, age-related differences depended on the use of encoding strategies: when participants employed strategies, older adults were less optimal than younger adults; when they did not, older adults were more optimal and calibrated than younger adults.^8^

Finally, Thomas et al. (2012, Experiment 1) showed that older adults were more conservative than younger adults when monitoring working memory for object locations (for a description of this study, see section JOL sensitivity in older adults). However, when JOLs concerned object identity or the bound identity and location, there were no age-related differences in JOL calibration. Moreover, equating encoding time in the follow-up experiments diminished age-related differences in JOL calibration, providing additional evidence in favor of age invariance in JOL calibration.

In summary, research on age-related differences in JOL calibration provides mixed results. Whereas some studies indicate that older adults are more optimal than younger adults, the majority of studies provide evidence for JOL suboptimality in older adulthood.

### Feeling-of-knowing (FOK)

Feelings-of-knowing **(**FOKs) are metacognitive experiences about the likelihood of successfully retrieving information in the future despite a current retrieval failure (Koriat, 1993, 2000; Metcalfe, 1986; Metcalfe et al., 1993). Empirically, FOKs are assessed by asking participants to judge the probability of recognizing the correct answer to a question amongst two or more alternatives. For example, a participant might first be asked to recall the name of the tallest mountain peak in South America (Mt. Aconcagua). If they cannot retrieve the correct name, the participant is then asked how likely they are to recognize the correct name from a list of several tall mountains. A high FOK indicates strong confidence in successful future recognition.^9^

Prominent theories of FOK consider this metacognitive experience to be the result of metacognitive inferential computations based on retrieval of partial recollection of the target information (Brewer et al., 2010; Koriat, 1993, 1995, 2000), the familiarity of the cue (e.g., question; Metcalfe et al., 1993; Schwartz & Metcalfe, 1992), or a combination of both (Koriat & Levy-Sadot, 2001). Consistent with this, research in younger adults showed that FOK is a reliable predictor of future retrieval (e.g., Koriat, 1993; Metcalfe et al., 1993; Sacher et al., 2009; Schwartz et al., 2014; Schwartz & Metcalfe, 1992).

### FOK sensitivity in older adults

Given the importance of FOK in memory retrieval, numerous studies have evaluated FOK metacognitive sensitivity (henceforth, FOK sensitivity) in older adults. In general, these studies fall into two categories: those that evaluated FOK in semantic memory retrieval (semantic FOK; see Table 4) and those that evaluated FOK in episodic memory retrieval (episodic FOK; see Table 5). Semantic FOK refers to judgments about the likelihood of recognizing unrecalled information from general knowledge, whereas episodic FOK refers to judgments about the likelihood of recognizing unrecalled information from recently learned events or experimentally studied associations. The experimental procedures to collect semantic and episodic FOK reports are quite similar, except that in episodic FOK tasks participants first learn the association between a cue (e.g., word, picture, face, etc.) and a target (e.g., word, name, etc.).

**Table 4:** Summary of studies that evaluated age-related differences in semantic feeling-of-knowing (FOK) sensitivity

Study	Experiment	Object-level task	Measure of interest	Result	Young		Old	
					*N*	Age, y	*N*	Age, y
Butterfield et al. (1988)	2	General-knowledge recognition	Gamma	Young = Old	54	18.6	36	67.7
Allen-Burge and Storandt (2000)	1	Rare-words definition recognition	Gamma	Young = Old	45	19.18	45	71.60
Lachman et al. (1979)	1	General-knowledge recognition	Probability of correct object-level response for each FOK Judgment category*	Young = Old	12	20.58	12	68.92†
Marquié and Huet (2000)	1	General-knowledge and computer-knowledge recognition	Gamma	Young = Old	22	21.95	22	68.41‡

*This is a very similar approach to the CAC curve method which is used to evaluate metacognitive calibration. Hence, we can argue that this study did not evaluate FOK sensitivity exclusively

† This study also included mid-life adults (*N *= 12, mean age = 49.92 years)

‡ This study also included mid-life adults (*N *= 22, mean age = 46.63 years)

**Table 5:** Summary of studies that evaluated age-related differences in episodic feeling-of-knowing (FOK) sensitivity

Study	Experiment	Object-level task	Measure of interest	Result	Young		Old	
					*N*	Age, y	*N*	Age, y
Sacher et al. (2015)	1	Cued-target recognition	Gamma Type 2 -ROC Curve	Young > Old	59	26.76	61	69.44
Souchay et al. (2000)	1	Cued-target recognition	Gamma	Young > Old	20	24.25	41	72.04
Perrotin et al. (2006)	1	Cued-target recognition	Gamma	Young > Old	40	24.27	62	72.52
Perrotin et al. (2008)	1	Cued-target recognition	Gamma	Young > Old			95	70.33†
Sacher et al. (2013)	1	Cued-target recognition	Gamma	Young (Control) > Young (DA at FOK) > Young (DA at encoding) = Old*	60	26.38	60	69.45
Thomas et al. (2011)	1	Cued-target recognition	Gamma	Young > Old	44	22.1	46	72.1
	2				47	18.7	37	70.1
	3				51	19.1	50	72.3
Souchay et al. (2007)	2	Cued-target recognition	Gamma	Young > Old	20	26.55	36	72.41
MacLaverty and Hertzog (2009)	1	Cued-target recognition	Gamma	Young = Old	206	20.6	200	69.50
Hertzog, Dunlosky, et al. (2010a)	1	Cued-target recognition	Gamma	Old (30-minutes) > Young = Old (2-days) 1-Repetition: Young > Old (2-days) 2-Repetitions: Young < Old (2-days) 4-Repetitions: Young = Old (2-days)	54	19.56	55 (30-minutes) 54 (2-days)	68.32 (30-minutes) 69.76 (2-days)
Eakin and Hertzog (2012)	1	Cued-target recognition	Gamma	Young = Old	51	19.58	42	68.09

*DA = divided attention

† This study only included participants ranging in age from 55 to 89 years

Studies evaluating semantic FOK sensitivity have typically reported age invariance. In the seminal study by Lachman et al. (1979), younger, middle-aged, and older adults demonstrated equivalent levels of semantic FOK sensitivity. Several subsequent studies further support these results. Allen-Burge and Storandt (2000, Experiment 1) presented participants with definitions of low-frequency words and asked them to recall the corresponding term. If participants could not recall the correct word, they rated their FOK on a 7-point scale and answered a series of questions about the unrecalled word (e.g., number of syllables, first letter). Next, participants completed a two-alternative forced-choice recognition task. Younger and older adults had equivalent γ coefficients, indicating age-invariance in semantic FOK sensitivity. Similar results are reported by Butterfield et al. (1988) and Marquié and Huet (2000).

Although these studies converge to suggest that semantic FOK sensitivity remains intact in aging, firm conclusions are limited by the metacognitive sensitivity measures. That is, it is quite possible that studies of semantic FOK did not find any evidence for age-related impairment or enhancement due to measures of metacognitive sensitivity used. This issue is especially important because semantic memory is one of the few cognitive functions that remains comparable between younger and older adults (Habeck et al., 2018; Nilsson, 2003; Park & Bischof, 2013).

In contrast to the largely age-invariant findings for semantic FOK, episodic FOK sensitivity has frequently been reported as reduced in older adults. Sacher et al. (2015) and Souchay et al. (2000) showed lower episodic FOK sensitivity in older relative to younger adults. In Souchay et al. (2000), participants studied 36 cue-target pairs and were later presented with each cue at test. When recall failed, participants made an FOK judgment and performed a memory recognition task. Results showed a lower γ coefficient in older than younger adults. Moreover, by asking participants to complete neuropsychological tests, Souchay et al. (2000) revealed that older adults’ lower episodic FOK sensitivity was related to reduced executive functioning. Similar results were reported by Perrotin et al. (2006, 2008), who found that impairments in set shifting, but not updating and inhibition, were associated with poorer episodic FOK sensitivity in older adults.

Two separate conclusions can be drawn from the results of these studies. First, age-related deficits in executive functions, such as set shifting, may lead to impairments of computations necessary for episodic FOK experiences. In line with this conclusion, Sacher et al. (2013) manipulated the divided attention in young adults by asking them to perform a secondary task either during the encoding phase or during the FOK judgment phase, allowing for a comparison of episodic FOK sensitivity across four groups: young adults with full attention (control group), young adults with divided attention at encoding, young adults with divided attention during the FOK-judgment phase, and older adults. Young adults with divided attention at encoding showed a comparable deficit in episodic FOK sensitivity to that of older adults. Moreover, young adults with divided attention during the FOK-judgment phase showed a lower episodic FOK sensitivity than the control group, but a better episodic FOK sensitivity than young adults with divided attention at encoding and old adults. These findings suggest that engaging younger adults in a secondary task mimics executive function deficits in older adults, which supports the results of earlier studies of episodic FOK in older adults.

Second, age differences in episodic FOK sensitivity may reflect impairments in the inferential computations used to evaluate partial retrieval cues and predict future recognition, which underlie episodic FOK. This interpretation, the inferential-deficit hypothesis, is compatible with prominent theories of FOK (Brewer et al., 2010; Koriat, 1993, 1995, 2000; Koriat & Levy-Sadot, 2001; Metcalfe et al., 1993; Schwartz & Metcalfe, 1992). Hence, age-related deficits cause a diminished capacity to evaluate the qualities of object-level information relevant for future retrieval. Based on this, older adults may be less capable of assessing the state of their episodic memory than younger adults.

Empirical support for this explanation is mixed. Thomas et al. (2011) found that, unlike younger participants, older adults were unable to use retrieved contextual partial information to make accurate episodic FOK predictions. In their study, participants learned 36 cue-target pairs, where each target varied in emotional valence (positive or negative). If recall failed, participants performed an episodic FOK task and tried to recall the valence of the unrecalled item before completing a recognition task. The results showed that younger adults had higher γ coefficients than older adults, reflecting better episodic FOK sensitivity. Younger adults appear to leverage contextual information more so than older adults, and to use these details to make accurate FOK predictions. Interestingly, when older adults were instructed to direct their attention towards the contextual information, the difference between the two groups was reduced (but did not disappear completely). These findings suggest that age-related reductions in episodic FOK sensitivity may be the result of impairments in the monitoring processes that evaluate episodic memory and deficits of executive functions that may, in turn, impair those monitoring processes.

On the other hand, Souchay et al. (2007) showed that lower episodic FOK sensitivity in older adults was associated with diminished recollection of contextual information, suggesting that object-level differences across age may impact metacognitive processes. Consistent with this interpretation, Hertzog, Dunlosky et al. (2010a) supported the memory-constraint hypothesis, proposing that age-related reductions in episodic FOK sensitivity is due to impaired memory processes and not the monitoring processes responsible for generating episodic FOK experiences. In addition, evidence for the memory-constraint hypothesis comes from MacLaverty and Hertzog (2009), who showed age-equivalent episodic FOK sensitivity. Participants studied 36 cue-target pairs and were randomly assigned to one of four between-subject conditions in a 2 × 2 design manipulating FOK timing (immediate vs. delayed) and recognition timing (immediate vs. delayed). Across all conditions, younger and older adults demonstrated an equivalent level of episodic FOK sensitivity. Neither the timing of the FOK judgment nor the timing of the recognition test significantly affected age differences in sensitivity. Other studies from the same lab support these results (Eakin & Hertzog, 2012; Hertzog, Dunlosky et al., 2010a). Using a repetition-delay paradigm, Hertzog, Dunlosky et al. (2010a) and Hertzog, Sinclair et al. (2010b) showed age-invariant episodic FOK sensitivity. In this study, younger and older adults studied 60 cue-target pairs assigned to three within-subject repetition levels (1-repetition, 2-repetition, and 4-repetition). After a delay that was adjusted across age groups (7-day delay for younger adults, 2-day delay for one group of older adults, and 30-min delay for another group of older adults), participants performed a memory recollection, FOK, and four-alternative forced-choice memory recognition tasks. Several aspects of the results supported the memory-constraint hypothesis. First, episodic FOK sensitivity did not differ between younger adults and older adults after a 2-day delay. Second, older adults with a 30-min delay performed better than younger adults and older adults with a 2-day delay. Third, the pattern of repetition effects indicated that when memory strength was enhanced, either through shorter delays or additional repetitions, age differences in episodic FOK sensitivity were attenuated or eliminated. Collectively, these results suggest that equating memory strength across age groups yields comparable levels of episodic FOK sensitivity, consistent with the memory-constraint hypothesis rather than the inferential-deficit hypothesis.

In summary, although some studies found an impairment of episodic FOK sensitivity in older adults, this age-related difference is reduced when memory strength is equated across older and younger adults. Therefore, age-related impairments of episodic FOK sensitivity may reflect underlying deficits in the memory processes that episodic FOK monitors.

Whether aging differentially affects semantic and episodic FOK sensitivities carries important theoretical implications for models of the metacognitive system. If aging only impacts episodic FOK, this would point to dissociable cognitive mechanisms supporting these two processes. However, the results of studies that equate episodic memory strength across age groups indicate that, similar to semantic FOK, episodic FOK may remain largely intact in older adults (see Table 6). Based on this, a direct comparison of age-related differences in semantic FOK and episodic FOK sensitivities using a within-subjects design is of critical importance.

**Table 6:** Summary of studies that evaluated age-related differences in judgment-of-learning (JOL) and feeling-of-knowing (FOK) sensitivities

Study	Experiment	Object-level task	Measure of interest	Result	Young		Old	
					*N*	Age, y	*N*	Age, y
Souchay et al. (2007)	1	Episodic cued-target recognition Semantic general-knowledge recognition	Gamma	Episodic Young > Old Semantic Young = Old	20	24.25	40	74.05
Eakin et al. (2014)	1	Episodic cued-face-name recognition Semantic celebrity face-name recognition	Gamma	Episodic Young = Old Semantic Young = Old	50	18.64	28	72.23
Morson et al. (2015)	1	Episodic general-knowledge recognition Semantic general-knowledge recognition	Gamma	Episodic Young > Old Semantic Young = Old	35	20.23	21	69.86

At least three studies have evaluated and compared semantic FOK and episodic FOK sensitivities within subjects and across younger and older adults. In Souchay et al. (2007, Experiment 1), participants answered general knowledge questions (e.g., “What is the subject of Magritte’s painting, *La* *Trahison*?") and studied cue-target pairs (e.g., Birthday-Pipe). Cleverly, the answers to the general knowledge questions and the target in the cue-target pairs were the same (e.g., “Pipe”) to ensure comparability between episodic and semantic FOK sensitivity. Results indicated that older adults had a lower episodic FOK but preserved semantic FOK sensitivity relative to younger adults.

General knowledge questions are inherently associated with the target answer, whereas the same level of inherent relatedness is often missing between cue-target pairs. Thus, age-related differences in episodic, but not semantic, FOK sensitivity may reflect differences between the two tasks in the level of cue-target relatedness. To test this, Eakin et al. (2014) used face stimuli as cues in both the semantic and the episodic memory task. In the semantic condition, participants recalled the names of famous celebrities; in the episodic condition, they first learned face-name associations with non-famous faces. In contrast to the results of Souchay et al. (2007), this study showed no age-related differences in either semantic or episodic FOK.

The discrepancy between Souchay et al. (2007) and Eakin et al. (2014) might be because of older adults’ efficient use of visual cues in Souchay et al.’s (2007) study. Morson et al. (2015) therefore used general knowledge questions in both semantic and episodic tasks. The semantic task was the same as previous studies of semantic FOK that used general knowledge questions (e.g., Souchay et al., 2007). The episodic task was quite similar to the semantic task, with only one difference: participants first studied the general knowledge questions and their correct answers before completing the FOK procedure. Here, older adults showed reduced γ coefficients in episodic, but not semantic, FOK sensitivity.

### FOK calibration in older adults

To date, only one study has evaluated semantic FOK calibration (Marquié & Huet, 2000). In this study, participants attempted to recall the correct answer to general knowledge questions or questions about computers. When recall failed, they provided an FOK rating. Results showed that older adults were more conservatively biased than younger participants for computer-related questions. Older and younger adults, however, had comparable levels of FOK calibration in reference to memory recognition of general-knowledge questions.

In contrast, three studies have evaluated age-related differences in episodic FOK calibration (see Table 7). Both Sacher et al. (2013) and Souchay et al. (2000) found reduced episodic FOK calibration in older adults than in younger adults. Moreover, Sacher et al. (2013) found that older adults are more conservatively biased in their FOK judgments. In contrast, Sacher et al. (2015), reported age-invariance in episodic FOK calibration. Hence, the literature depicts a contrasting picture regarding FOK calibration, which may be due to the measures used to estimate FOK calibration and the influences of object-level sensitivity and calibration in cognitive domains that show differential patterns of decline in aging.

**Table 7:** Summary of studies that evaluated age-related differences in feeling-of-knowing (FOK) calibration

Study	Experiment	Object-level task	Measure of interest	Result	Young		Old	
					*N*	Age, y	*N*	Age, y
Marquié and Huet (2000)	1	General-knowledge recognition	Average JOL Ratings*	Young = Old	22	21.95	22	68.41
		Computer-knowledge recognition		Older adults Conservative bias				
Sacher et al. (2013)	1	Cued-target recognition	Over-/under-confidence index	Older adults Conservative bias	60	26.38	60	69.45
Souchay et al. (2000)	1	Cued-target recognition	Hamann Coefficient	Older adults Lower calibration	20	24.25	41	72.04
Sacher et al. (2015)	1	Cued-target recognition	Calibration Index	Young = Old	59	26.76	61	69.44

^*^In this study, younger adults (DA at encoding) and older adults were similar to each other and more conservative than younger adults in the control group

### Comparing age-related changes in FOK and JOL sensitivity and calibration

In addition to evaluating age-related changes in metacognitive sensitivity and calibration of JOL and FOK separately, Souchay and Isingrini (2012) compared age effects in FOK sensitivity (γ coefficient) and calibration (proportion of high-confidence JOL and FOK ratings). In this study, participants provided delayed JOLs and episodic FOKs in separate blocks using procedures comparable to prior studies. Older adults demonstrated reduced FOK sensitivity compared to younger adults, whereas delayed-JOL sensitivity did not differ between age groups. Moreover, older adults were more liberally biased in the JOL task. There was no significant age-related difference in FOK calibration.

### Tip-of-the-tongue (TOT)

Tip-of-the-tongue (TOT) is a metacognitive feeling of imminent retrieval of an item from memory despite recall failure (Brown, 1991; Brown & McNeill, 1966; Schwartz & Metcalfe, 2011; Schwartz & Pournaghdali, 2020). For example, if an individual fails to recall the name of the tallest mountain peak in South America (Mt. Aconcagua), they may have the urgent feeling that they can remember the name at any moment. Research indicates that TOTs occur across cultures (Brennen et al., 2007; Schwartz, 1999) and throughout the lifespan (Salthouse & Mandell, 2013). Studies of TOT in aging focused on factors that may influence the frequency of TOTs (for a review, see Schwartz & Frazier, 2005). The main finding of these studies is an age-related increase in the frequency of TOTs, where older adults experience a higher number (Huijbers et al., 2017; Salthouse & Mandell, 2013). However, only a handful of studies evaluated age-related differences in the accuracy of TOTs (e.g., Gollan & Brown, 2006; Maylor, 1990; White & Abrams, 2002). Such studies did not systematically evaluate TOT sensitivity and did not use the common statistical approaches used to evaluate metacognitive sensitivity. Additionally, the results of these studies are inconclusive, with some showing age-related differences in TOT sensitivity (e.g., Maylor, 1990), and other studies failing to find such an effect (e.g., Abrams et al., 2007). Therefore, given the frequency of TOTs in older adults, evaluating age-related differences in TOT sensitivity and calibration is an area for growth in the field with both theoretical and practical implications, as this can inform models of metacognitive monitoring in aging and clarify whether older adults can use TOT experiences to guide retrieval strategies or compensatory behaviors in everyday communication.

### Ease-of-learning

Ease-of-learning (EOL) is a metacognitive estimation of how difficult a learning task will be (Jemstedt et al., 2017, 2018; Kornell et al., 2011). For example, before studying a list of words, participants may be asked to estimate how challenging they expect the task to be. Older adults often overestimate difficulty when learning new things or acquiring new skills, a phenomenon referred to as the “negative bias in aging.” This tendency has been linked to changes in metacognitive calibration and broader age-related cognitive changes. One potential cause is decline in fluid intelligence, which involves problem solving, reasoning, and novel learning (Kievit et al., 2014; Manard et al., 2014). Older adults who are more metacognitively aware of such declines in fluid intelligence may assume that learning new things will be more difficult (Hess, 2014). Age-related changes in emotional processing could result in more salient or memorable recollections of challenging learning experiences (Labouvie-Vief et al., 2010). This selective memory can lead to an overestimation of the difficulty of new learning. Further, as people age, they may become more risk-averse and concerned about making mistakes or failing, where the fear of failure can lead to a perception that learning something new is harder than it actually is. Finally, older adults may have fewer opportunities to engage with new technologies and experiences, leading to a perception that the world is changing rapidly and that learning new things is more difficult (Salthouse, 2006). Clarifying whether and how EOLs change with age is an important direction for future investigation.

### Post-decision confidence

Post-decision confidence (henceforth, confidence) judgments are retrospective metacognitive estimates of the accuracy of one’s cognitive and perceptual decisions (Grimaldi et al., 2015; Mamassian, 2016; Rahnev et al., 2020). In contrast with other metacognitive judgments such as JOLs, confidence judgments are collected after an object-level decision has been made. For example, after deciding whether the face-name pair is intact or not, a participant reports how confident they are that their decision was correct. Identifying the underlying neurocognitive mechanism(s) of confidence is a matter of extensive investigation, and to this end, many computational models have been introduced (e.g., Fleming & Daw, 2017; Kiani et al., 2014; Maniscalco et al., 2021; Maniscalco & Lau, 2016; Pleskac & Busemeyer, 2010; Pouget et al., 2016; Sanders et al., 2016; Shekhar & Rahnev, 2021). Empirically, confidence is usually, but not always, a reliable predictor of the accuracy of object-level decisions in younger adults.

A critical obstacle, which may slow down the progress to study possible age-related differences in confidence sensitivity and calibration, is the heterogeneity of object-level decisions that confidence judgments are supposed to evaluate. Whereas other metacognitive judgments evaluate a limited set of object-level decisions, confidence judgments can evaluate the accuracy of almost all types of cognitive or perceptual decisions. For example, FOKs are directed toward semantic and episodic memory recognition in the absence of successful recall. In contrast, confidence judgments can be applied to perceptual tasks (e.g., motion discrimination, facial identification, and color discrimination; Pournaghdali et al., 2023; Rausch et al., 2015), memory tasks (e.g., episodic recall, semantic recognition in the absence of successful memory recall, and eyewitness identification; Chua et al., 2012; Delay & Wixted, 2021; Metcalfe et al., 2023; Wixted & Wells, 2017), problem-solving tasks (e.g., verbal and mathematical problems; Ackerman & Zalmanov, 2012), and motor decisions (Charles et al., 2020). Therefore, confidence judgments can be categorized based on the object-level decisions that they are evaluating. This classification results in a variety of confidence, including memory confidence, perceptual confidence, motor movement confidence, and so on. Here, we primarily focus on memory and perceptual confidence judgments, as these account for the majority of studies examining confidence in older adults.

### Confidence evaluation of memory decisions

#### Memory confidence sensitivity

Research on confidence evaluations of semantic memory recollection (hereafter, semantic confidence; see Table 8 for a summary) indicates that older adults have a comparable or, under certain conditions, better confidence sensitivity than younger adults (Dodson et al., 2007; Perlmutter, 1978; Pliske & Mutter, 1996; also see Marquié & Huet, 2000). This may not be surprising, given robust evidence that semantic memory is relatively preserved, and may even increase, with age (Brickman & Stern, 2009; Nilsson, 2003). Pliske and Mutter (1996) showed that older adults had higher semantic confidence sensitivity when evaluating the accuracy of memory recollection of general knowledge questions, as measured by γ correlation. In contrast, Perlmutter (1978) asked participants to judge the truthfulness of various statements and to rate their confidence in each decision. Here, older and younger adults had comparable semantic confidence sensitivity. Similarly, Dodson et al. (2007, Experiment 2) showed that older adults’ semantic confidence sensitivity was comparable to that of younger adults for easy and moderately hard general knowledge questions. Notably, when evaluating difficult general knowledge questions, older adults showed better semantic confidence sensitivity than younger adults. In summary, many studies have found that older adults have equivalent, or sometimes better, semantic confidence than younger adults.

**Table 8:** Summary of studies that evaluated age-related differences in memory confidence sensitivity

Study	Experiment	Object-level task	Measure of interest	Result	Young		Old	
					*N*	Age, y	*N*	Age, y
Dodson et al. (2007)	1	Old/new recognition	Gamma	Young = Old	24 (young) 24 (young delay)	21.3 (young) 18.5 (young delay)	24	67.3
		Source memory recognition	Gamma	Young > Old				
	2	Cued-recall recognition	Gamma	Young = Old	36	18.6	18	67.3
		General-knowledge recognition	Gamma	Old > Young for difficult Qs Young = Old for moderate and easy Qs				
Hertzog et al. (2021)	1	Cued-recall Recognition	Gamma	Young = Old	120		56	70.52
Zakrzewski et al. (2021)	1	Yes/no item recognition Yes/no associative recognition	Gamma Meta-d’ M-ratio	Young = Old	24	19.38	25	73.3
Wong et al. (2012)	1	2AFC memory recognition	Gamma	Young > Old	112	19.69	56	77.72
Kelley and Sahakyan (2003)	1	Word-pair cued recall	Gamma	Young > Old	60	18.8	60	72.8
Perlmutter (1978)	1	Old/new word recognition	Mean confidence for correct vs. incorrect trials	Young = Old	32	23	32	62
		True/false fact verification task	Mean confidence for correct vs. incorrect trials	Young = Old				
Pliske and Mutter (1996)	1	General knowledge questions	Gamma	Young < Old	22	21.45	22	68.64
Kuhlmann and Boywitt (2016)	1	Source memory	Mean confidence for correct vs. incorrect trials	Young > Old	49	20.98	43	72.07
	2	Source memory	Mean confidence for correct vs. incorrect trials	Young > Old	40	22.05	40	70.73
Hines et al. (2009)	1	Word-pair cued recall	Gamma	Young = Old	39	21.8	35	66.8
Wahlheim et al. (2017)	1	Words free recall	Difference between correct and incorrect metacognitive judgments	Younger > Old	30	18.97	30	76.97
Marquié and Huet (2000)	1	General-knowledge and computer-knowledge recognition	Gamma	Young = Old	22	21.95	22	68.41
Pupillo et al. (2024)	1	Old/new associative recognition	Meta-d’ M-ratio	Younger > Old	397	28.32	1522	67.56

In a seminal study investigating metacognitive sensitivity of confidence evaluations of episodic recollection, or episodic confidence, Perlmutter (1978) found comparable results between older and younger adults using an old/new memory recognition paradigm. Further studies of episodic confidence employing old/new memory recognition and cued recall similarly found age-invariant confidence sensitivity (Dodson et al., 2007; Hines et al., 2009). More recently, Hertzog et al. (2021) examined episodic confidence using a category-based recognitive task while equating memory performance across age groups via retention interval manipulation. Participants studied 40 different sets of four semantically related nouns (e.g., fruits), with all four nouns presented in each set belonging to the same category (e.g., fruit category), with one target noun per set presented in red for memorization. Older adults and one group of younger adults performed the memory recognition task after a 2-day delay, while another group of younger adults performed the recognition task after a 7-day delay. In the recognition phase, participants selected the target from five alternatives, with two studied lures and two novel lures. Results showed that older adults had comparable episodic confidence sensitivity to both younger adult groups, alongside similar recognition performance. Zakrzewski et al. (2021) replicated these findings of age-invariant episodic confidence sensitivity using signal detection theory-based measures of metacognitive sensitivity, such as meta-d′ and M-ratio (see Online Supplementary Material, *How to Measure Metacognitive Sensitivity and Calibration*), as well as γ coefficient. In this study, older and younger adults studied a series of word pairs. In the testing phase, participants judged whether single words or word pairs had appeared during the study phase and rated their confidence in the accuracy of their decision. Contrasting meta-d′, M-ratio, and γ coefficient between the two age groups revealed no significant difference.

In contrast, other studies have found evidence in support of age-related impairments in episodic confidence sensitivity, especially when confidence is evaluating source memory recognition (Dodson et al., 2007, Experiment 1; Kuhlmann & Boywitt, 2016; Wong et al., 2012; also see Wahlheim et al., 2017). For example, Kelley and Sahakyan (2003) asked older and younger adult participants to study 60 word pairs and test their memory recollection using stem completion (participants were presented with the cue word and the first three letters of the target and were asked to recall or guess the target), providing a confidence judgment for each response. Older adults, on average, had lower γ coefficients than younger adults, indicating reduced episodic confidence sensitivity. Dodson et al. (2007) also observed age-related impairments in confidence sensitivity for source memory recognition but not for old/new recognition tasks (for similar results regarding age-related impairment of confidence evaluation for source memory, see Kuhlmann & Boywitt, 2016). More recently, Pupillo et al. (2024) reported reduced episodic confidence sensitivity in older adults using an associative recognition task that required discrimination between intact, rearranged, and new face-profession pairs and measured by the M-ratio. Age-related deficits were particularly pronounced for rearranged pairs, which demand additional monitoring and rejection of false associations.

Wong et al. (2012) extended these findings using a two-alternative forced-choice memory recognition task. Participants first made pleasantness judgments on object names in black ink, followed by two encoding blocks. In one block, participants were presented with either colored or black-and-white pictures of half of the objects. In the other block, participants were presented with the names of the other half of the objects in red or blue font. Older adults and one group of younger adults studied the presented stimuli under full attention, whereas a second group of younger adults studied the presented stimuli while performing a secondary attention task. At test, participants completed forced-choice memory recognition tasks about either the pictures or the fonts (such as identifying which object has been presented as a colored image or in blue font) and rated their confidence. Episodic confidence sensitivity was lower in older than younger adults for both picture and font source tasks. Critically, even when performance was equated by comparing older adults with younger adults in the divided attention group, older adults showed lower confidence sensitivity.

In summary, the evidence regarding age-related differences in episodic confidence sensitivity is task dependent. Older adults’ confidence sensitivity is comparable when evaluating memory recognition in the form of old/new recognition or cued recall, but impairments emerge when judgments concern source memory. This pattern implies that episodic confidence sensitivity deficits in older adulthood may be more pronounced for source memory than for other types of episodic memory recollection.

It should be noted that studies reporting age-related impairments in episodic confidence sensitivity did not rule out the possible influence of age-related differences in memory sensitivity and accuracy. Moreover, the heterogeneity of measures used to evaluate confidence sensitivity may contribute to inconsistencies regarding age-related differences in episodic confidence sensitivity. Future studies should examine multiple memory tasks within the same sample to understand the impact of aging on episodic confidence sensitivity.

### Memory confidence calibration

Several studies have evaluated memory confidence calibration in older and younger adults (see Table 9); however, these studies paint an inconclusive picture regarding age-related differences. With respect to semantic confidence, for example, Pliske and Mutter (1996) found no age-related differences in calibration (see also Hansson et al., 2008), whereas Dodson et al. (2007) found better calibration scores in older than younger adults.

**Table 9:** Summary of studies that evaluated age-related differences in memory confidence calibration

Study	Experiment	Object-level task	Measure of interest	Result	Young		Old	
					*N*	Age, y	*N*	Age, y
Dodson et al. (2007)	1	Old/new recognition	Calibration Index	Younger = Older	24 (young) 24 (young delay)	21.3 (young) 18.5 (young delay)	24	67.3
		Source memory recognition	Calibration Index	Older adults Less calibrated				
	2	Cued-recall Recognition	Calibration Index	Older adults Less calibrated	36	18.6	18	67.3
		General-knowledge recognition	Calibration Index	Older adults Less calibrated				
Wong et al. (2012)	1	2AFC memory recognition	Calibration Index Confidence–accuracy discrimination score	Younger = Older*	112	19.69	56	77.72
Kelley and Sahakyan (2003)	1	Word-pair cued recall	Calibration Index	Older adults Less calibrated**	60	18.8	60	72.8
Pliske and Mutter (1996)	1	General knowledge questions	Calibration Index	Younger = Older	22	21.45	22	68.64
Chua et al. (2009)	1	Face-name recall	Hamman Index	Older adults Less calibrated***	16	Ages 21–29	16	Ages 66–81

*In this study, when object-level performance was matched between the two age-groups, the difference in calibration error score, but not discrimination score, disappeared

**In this study, older adults had lower calibration only for the deceptive, but not control, items

***In this study, older adults were generally less calibrated. However, older and younger adults had a comparable level of confidence calibration in the low level of confidence, and older adults were less calibrated when using a higher level of confidence

Regarding episodic confidence, Dodson et al. (2007) found lower confidence calibration in older adults when evaluating source memory and cue-recall recognition decisions. In contrast, there was no significant difference in confidence calibration between the two age groups when confidence was evaluating old/new recognition. In addition, Kelley and Sahakyan (2003) showed that older adults have lower calibration for word pairs that can cause interference (e.g., “nurse-dollar,” with “doctor” as an interfering alternative), but comparable calibration to younger adults for word pairs without interference (e.g., “clock-dollar”). These results, like those of episodic FOK (e.g., Sacher et al., 2013; Souchay et al., 2000), implicate the role of age-related changes in executive function, particularly inhibitory processes (see Eich et al., 2018; Gurat & Medula, 2016; Hasher et al., 1991; Hasher & Zacks, 1988), in metacognitive processes.

Moreover, Chua et al. (2009) showed that older adults have lower episodic confidence calibration than younger adults in a face-name associative memory task. Participants studied novel face-name pairs and later recalled the associated name for each face, followed by confidence ratings. The results indicated lower episodic confidence calibration in older than younger adults. Likewise, Wong et al. (2012) found lower confidence calibration in older adults; however, when object-level performance was equated, age-related differences in confidence calibration disappeared.

### Confidence evaluation of perceptual decisions

Extensive research has shown that confidence is a reliable indicator of the accuracy of perceptual decisions (e.g., Allen et al., 2017; McCurdy et al., 2013; Morales et al., 2018). Therefore, many studies have investigated conditions that may impact confidence evaluations of perceptual decisions, or perceptual confidence, and the underlying neurocomputational mechanisms. Surprisingly, only a handful of studies have evaluated perceptual confidence in older adults.

### Perceptual confidence sensitivity

Research on age-related differences in perceptual confidence depicts a mixed picture, particularly with perceptual confidence sensitivity (see Table 10). In a cross-sectional study, Filippi et al. (2020) showed no age-related differences. Participants, spanning ages 7 to 80 years, were presented with two same-sized circles, one on each side of the visual field and each containing a random number of dots. Participants indicated which circle contained more dots and rated their confidence in their decision. Perceptual confidence sensitivity did not vary as a function of age. Using a similar task, Culot et al. (2023) compared older adults with major depression disorder with older and younger adults with no history of depression. Comparison of metacognitive efficiency in perceptual confidence using the M-ratio revealed age-invariance in perceptual confidence efficiency. In both studies, confidence was assessed with separate post-decision ratings.

**Table 10:** Summary of studies that evaluated age-related differences in perceptual confidence sensitivity

Study	Experiment	Object-level task	Measure of interest	Result	Young		Old	
					*N*	Age, y	*N*	Age, y
Filippi et al. (2020)	1	2AFC	M-ratio	Younger = Older	78	25.3	50	68.1†
Culot et al. (2023)	1	2AFC	M-ratio	Younger = Older	20	23.47	20	75‡
Klever et al. (2022)	1	2AFC	Confidence Modulation Index	Younger > Older	30	24.6	30	68.8
Overhoff et al. (2021)	1	Erikson flanker task	Phi Correlation	Younger > Older				‡‡

† This study also included mid-life adults (*N *= 42, mean age = 43.9 years)

‡ This study also included older adult patients with Major Depression Disorder (MDD) (*N *= 17, mean age = 74.88 years)

‡‡ This study included healthy adults ranging in age from 20 to 76 years (*N *= 65, mean age = 45.5 years), with an approximately uniform age distribution and a minimum of ten participants per decade

In contrast, studies reporting age-related declines have either used alternative confidence designs or had increased task demands. Klever et al. (2022) presented participants with two consecutive perceptual tasks. In each task, participants completed contrast discrimination judgments between two Gabor patches. Rather than providing separate confidence ratings, participants indicated which of the two decisions they felt more confident about. Older adults, on average, had lower perceptual confidence sensitivity than younger adults. Importantly, comparative confidence judgments reduce the influence of response biases on metacognitive estimates and mitigate external implications from the use of a confidence scale. As such, this approach may provide an estimate of confidence sensitivity that is more resistant to bias.

Similarly, Overhoff et al. (2021) also provided evidence for age-related impairments in perceptual confidence sensitivity with increased task complexity. In this cross-sectional study, participants ranging in age from 20 to 81 years classified the color of a central square by congruent or incongruent distractor squares. After making each decision, participants rated their confidence in their decision. Perceptual confidence sensitivity decreased with age, and notably, the effect was not influenced by age-related decline in task performance. This suggests that under conditions of increased executive or attentional demands, age-related differences in confidence sensitivity may become more apparent.

In summary, discrepancies across studies likely reflect methodological contributions from the cognitive demands of the perceptual task and the format used to quantify confidence. Studies reporting age invariance used relatively simple perceptual tasks with minimal memory load or interference. In contrast, studies observing age-related impairments have used tasks that either require comparison across multiple perceptual decisions (Klever et al., 2022) or involve perceptual interference and increased executive demands (Overhoff et al., 2021). Hence, this raises the possibility that age-related differences in perceptual sensitivity emerge primarily when older adults are required to perform a more complex perceptual or confidence task that relies on intact executive processes. In such cases, age-related differences in perceptual confidence sensitivity may reflect the lack of sufficient executive or perceptual processing resources needed for an efficient confidence evaluation, rather than impaired confidence sensitivity in older adults. In addition, rating-based measures may be more susceptible to response biases, potentially obscuring age-related differences, whereas comparative confidence paradigms may provide a more constrained estimate of confidence discrimination. Thus, age effects in perceptual confidence sensitivity may emerge depending on task complexity and design.

### Confidence sensitivity: Perceptual versus memory

At least two cross-sectional studies have directly compared perceptual and memory confidence sensitivities in older and younger adults (see Table 11). Palmer et al. (2014) examined both domains using a two-interval forced-choice perceptual task, in which participants indicated whether the target stimulus was presented in the first or the second interval, and a two-alternative forced-choice memory recognition task, in which they discriminated old words from novel ones. After each decision, participants rated their confidence in the accuracy of their decisions. Metacognitive efficiency, quantified using the M-ratio, revealed an age-related impairment in perceptual confidence efficiency but not in memory confidence efficiency. In contrast, McWilliams et al. (2023) found evidence in support of age-invariance in both perceptual and memory confidence efficiency, also measured by M-ratio. Their memory task was an old/new memory discrimination of previously studied objects from two alternatives. The perceptual task was a two-alternative forced-choice task in which participants indicated whether there were more blue or red colored dots on the screen. Under these conditions, no significant age differences emerged in either domain. Similarly, Meunier-Duperray et al. (2025) directly compared domains within a single lifespan sample using a meta-d′ framework. While they observed age-related reductions in episodic confidence sensitivity and increases in semantic confidence sensitivity, perceptual confidence sensitivity did not differ as a function of age, further suggesting that aging effects on metacognition are domain dependent rather than global.

**Table 11:** Summary of studies that evaluated age-related differences in memory and perceptual confidence sensitivity

Study	Experiment	Object-level task	Measure of interest	Result	Young		Old	
					*N*	Age, y	*N*	Age, y
Palmer et al. (2014)	1	2AFC (Perceptual)	M-ratio	Young > Old				†
		2AFC (Memory)		Younger = Old				
McWilliams et al. (2023)	1	2AFC (Perceptual)	M-ratio	Young = Old				‡
		Old/New memory discrimination task		Young = Old				
Meunier-Duperray et al. (2025)	1	2AFC (Perceptual)	Meta-d’	Young = Old				‡‡
		Old/new recognition (episodic memory)		Young > Old				
		Multiple-choice general-knowledge (semantic memory)		Young < Old				

† This study included healthy adults ranging in age from 18 to 84 years (*N *= 60, mean age = 40.28 years)

‡ This study included healthy adults in six age groups (*N *= 50, age groups = 18–27, 28–37, 38–47, 48–57, 58–67, and 68 years and over)

‡‡ This study included healthy adults ranging in age from 18 to 79 years (*N *= 442, mean age = 42.40 years)

In summary, investigations across domains converge in showing that age-related differences in confidence sensitivity are not uniformly expressed across cognitive systems. Memory confidence sensitivity often appears preserved in standard recognition paradigms, though episodic impairments may emerge depending on task demands, whereas perceptual confidence findings are more variable across paradigms. One reason for this discrepancy is the nature of the perceptual task. Whereas Palmer et al. (2014) used a two-interval forced-choice perceptual detection task, McWilliams et al. (2023) and Meunier-Duperray et al. (2025) used a two-alternative forced-choice discrimination task. Detection and discrimination rely on distinct processes and information, and the confidence evaluations of these two tasks might be based on different computations (e.g., Hol & Treue, 2001; Sagi & Julesz, 1984). For instance, one of these computations may be affected by age-related differences, but not the other (e.g., Beard et al., 1994; McKendrick et al., 2018). Therefore, direct comparisons across studies using different perceptual paradigms may be misleading (also see Peters et al., 2016). Future studies should systematically compare detection and discrimination tasks within the same sample.

## Concluding remarks

The aim of this review was to synthesize the current literature on aging and its impact on metacognitive sensitivity and calibration across different metacognitive experiences. We began by outlining the primary measures of metacognitive sensitivity and metacognitive calibration, and reviewed the current literature based on two criteria:

1. Whether resulting age-related differences in one construct (e.g., metacognitive calibration) could be explained by age-related differences in the other (e.g., metacognitive sensitivity), and
2. Whether resulting age-related differences in either construct could be explained by age-related differences in object-level processes.

What conclusions can be drawn from the existing evidence? Regarding JOL, FOK, and confidence, the current literature provides a mixed view. These inconsistencies may derive from several factors, two of which are the focus of this review. First, the measures used to evaluate metacognitive sensitivity and metacognitive calibration may overestimate or underestimate actual metacognitive sensitivity in older and younger adults. As a result, subtle age-related differences in metacognitive sensitivity or calibration may go undetected. For example, γ correlation frequently used to assess semantic FOK sensitivity can be influenced by metacognitive bias, which may differ across age groups and complicate the detection of small but meaningful age-related effects.

Beyond measurement limitations, variability in results across studies may be due to interacting sources of heterogeneity, including differences in the tasks used, sample characteristics, and the specific metacognitive measure employed (e.g., γ correlation vs. meta-d′). These factors are rarely held constant across studies, making direct comparison difficult. Consensus on gold standard paradigms and model-based measures that are less susceptible to object-level confounds is needed.

One reason for the limitation of many measures of metacognitive sensitivity and bias is their inability to exclude object-level effects in task performance from the metacognitive construct they are estimating. Aging is known to impact both object-level sensitivity and bias (e.g., Devaluez et al., 2023), and such changes may influence estimates of metacognitive sensitivity and calibration (e.g., Galvin et al., 2003; Rahnev, 2023). Hence, when age differences in metacognitive indices are observed, it is often unclear whether they reflect changes in metacognitive processing or are secondary to differences in object-level processing.

This issue is particularly relevant to findings of FOK sensitivity. Although Devaluez et al.’s (2023) meta-analysis quantified age-related differences in FOK, aggregate effect sizes cannot determine whether such differences stem from metacognitive-level alterations or from underlying memory strength. Addressing this question requires examining moderator patterns across studies – for example, whether equating memory performance attenuates age effects – rather than relying solely on mean effect estimates.

In the semantic domain, studies that evaluated FOK and confidence sensitivities generally show age equivalence in memory recollection performance. These results are consistent with studies specifically evaluating semantic recollection in older adults that show preserved function of semantic memory in older adulthood. Based on this, preserved semantic FOK and confidence reflect intact metacognitive monitoring of semantic information in underlying neurocognitive mechanisms (e.g., Allen-Burge & Storandt, 2000, Experiment 1; Dodson et al., 2007; Perlmutter, 1978). However, this conclusion may occlude an important aspect of metacognitive monitoring: In most studies of semantic FOK and semantic confidence in older adults, memory task difficulty was not systematically manipulated. That is, the unrecalled information often retained high accessibility and were relatively easy to recognize later. Systematic manipulation of item difficulty in both older and younger adults would provide a more rigorous test of age-related differences in semantic FOK or confidence sensitivity. A similar approach would be informative for JOL, episodic FOK, and episodic confidence.

Another issue to consider is the relationship between individual, item-level metacognitive judgments and broader global self-evaluations. In healthy young adults, fluctuations in item-level confidence contribute to global self-performance estimates above and beyond objective task accuracy, indicating that global self-evaluations are partially derived from accumulated confidence information (Rouault et al., 2019). However, the mapping between local monitoring processes and global self-appraisal does not appear to be uniform across populations. For example, individuals with Alzheimer’s disease (AD) were shown to have relatively preserved trial-level confidence-accuracy associations, but exhibited impaired global awareness of memory decline (Gallo et al., 2012). These findings suggest that local metacognitive sensitivity and global self-evaluative insight may be dissociable. Clarifying the relationship between these processes is particularly relevant in the context of healthy aging and SCD.

An additional layer of complexity is introduced by the idea of “meta-metacognition,” the capacity to reflect on the accuracy of one’s own monitoring processes (Dunlosky et al., 2005; Recht et al., 2022). If individuals differ not only in how they monitor performance but also in how well they evaluate that monitoring, age-related changes in this recursive process may be particularly revealing. Examining such changes could provide insight into SCD, especially in cases where reported difficulties exceed – or fall short of – objective performance patterns.

A further issue concerns common reliance on null hypothesis significance testing to infer age equivalence in metacognitive sensitivity. In many cases, non-significant results are interpreted as evidence of preservation, although such findings do not necessarily distinguish between true invariance and insufficient statistical power. Reporting Bayes factors in favor of the null (BF₀₁), where possible, would offer a more informative basis for evaluating claims of age invariance. This distinction is especially important given the possibility that some measures may lack the precision required to detect subtle age-related differences. Accordingly, non-significant findings should be interpreted with caution, and stronger evidential standards should be encouraged in future research.

This review focused on item-based metacognitive experiences and judgments, which are judgments in reference to a specific memory or object-level decision. However, metacognitive monitoring also encompasses global evaluations to one’s overall object-level capabilities. For example, individuals may provide global JOLs estimating the probability of correct recollection of a set of studied items (e.g., Connor et al., 1997; Händel et al., 2020). They may also rate how their memory compares to someone else or to their past performance. Such metacognitive judgments form the basis of subjective cognitive decline (SCD; Jessen et al., 2014), defined as the perception of cognitive deterioration prior to the emergence of objective clinical impairment (also see Chapman et al., 2021). SCD is considered one of the earliest symptoms of AD (see Jack et al., 2024), is associated with AD biomarkers (Amariglio et al., 2012, 2015; Buckley et al., 2019; Perrotin et al., 2012), and predicts progression to mild cognitive impairment and dementia (e.g., Chapman et al., 2023; Jessen et al., 2010; Rabin et al., 2017; van Harten et al., 2018; also see Pike et al., 2022).

Although a comprehensive review of metacognition in neurodegenerative diseases such as AD and related disorders is beyond the present scope, metacognitive functioning and variability therein plays a central role in the detection, experience, and management of cognitive decline (Cosentino, 2014; Sunderaraman & Cosentino, 2017). Clarifying how aging affects both item-level and global metacognitive processes is therefore not only theoretically important but also clinically consequential.

## Supplementary Information

Below is the link to the electronic supplementary material.

Supplementary file1 (PDF 172 KB)

## Data Availability

Not applicable.

## Footnotes

1 This gamma coefficient was estimated based on the assumption that it mimics gamma coefficient when using old/new recognition.

2 This gamma coefficient was estimated to assess the influence of a more conservative and restricted memory recollection on JOL sensitivity.

3 The new names were also composed of actors from 1950 s and 1990s.

4 For a description of type-2 ROC curve, see the Online Supplementary Material.

5 When gamma coefficient was estimated separately for 1950 s and 1990 s names, there was no age-related difference in JOL sensitivity.

6 A CAC curve, as a measure of metacognitive calibration, is a plot representing proportion of correct object-level responses in the y-axis against different ratings of a metacognitive judgment (e.g., 0, 10, 20, 30, …, 90, 100% confidence ratings) in the x-axis. A complete diagonal line of a CAC curve represents complete metacognitive calibration. Points or lines above the diagonal line representing under-confidence and points or lines below diagonal line representing over-confidence (see Online Supplementary Material, section *Measurements of metacognitive calibration*)*.*

7 Robinson et al. (2006) did not perform statistical analysis of calibration. The only mentioned that older adults were over-confident (liberal bias), whereas the younger adults were under-confident (conservative bias). Comparing the level of deviance from the optimal level, we may assume that older adult (10% and 4% deviance from optimal in Experiments 1 and 2, respectively) were closer to the optimal level than younger adults (19% and 24% deviance from optimal in Experiments 1 and 2, respectively; see Robinson et al., 2006, Table 2). Hence, in the absence of a statistical comparison of calibration between younger and older adults, we should interpret Robinson et al.’s results with caution.

8 Older adults’ JOL sensitivity did not interact with the use of encoding strategy in this study.

9 Participants usually make a FOK judgment when their recall fails. However, depending on the goals of a study, we can ask participants to make FOK judgments for both recalled and unrecalled information (Schwartz et al., 2016).
