# Supplementary material for: Metacognition and metacognitive monitoring in aging
Source: Psychon Bull Rev. 2026 Aug 4;33(6):205. doi: 10.3758/s13423-026-02968-7 (PMC13437673; doi:10.3758/s13423-026-02968-7)
Supplement: Supplementary file 1 [file 13423_2026_2968_MOESM1_ESM.pdf]

Docusign Envelope ID: DDCDB67E-9EAA-4BE0-80DF-2D3EED6F5448

Dear Stephanie and Ali,

I am writing to request your formal approval to add Gloria Cheng as a co-author on the revised version of our manuscript. Gloria provided substantial assistance during the revision process, particularly with the review and refinement of the manuscript.

Because the number of authors listed in the revised submission differs from the original submission, the journal asked for written confirmation from all co-authors, agreeing to this change in authorship.

If you are in agreement, please sign below so that I include this documentation with our resubmission.

Thank you both!

Teal

DocuSigned by:
1FE2C61B3CC7467...

Signed by:
Gloria Cheng
1C1BD522440D4AA...
Gloria Cheng 3/3/2026

Signed by:
Ali Pournaghdali
4511ADACE25F473...
Ali Pournaghdali 3/3/2026

Signed by:
Stephanie Cosentino
AB8C530581FB4A4...
Stephanie Cosentino 3/3/2026
